# Surgical Approaches to Optic Nerve Decompression in Osteopetrosis: A Review of Endoscopic Endonasal and Transcaruncular Techniques

**DOI:** 10.1055/a-2544-3435

**Published:** 2025-03-20

**Authors:** Rita Maria Jalkh, Patrick Abou Raji Feghali, Ghena Lababidi, Houssein Darwish, Zeina Korban

**Affiliations:** 1 Department of Otolaryngology, American University of Beirut Medical Center, Beirut, Lebanon; 2 Department of Neurosurgery, American University of Beirut Medical Center, Beirut, Lebanon

**Keywords:** osteopetrosis, optic nerve decompression, endoscopic endonasal approach, transcaruncular, pediatric

## Abstract

Osteopetrosis is a rare genetic disorder characterized by impaired osteoclast function and excessive bone density, often leading to compressive optic neuropathy due to bony overgrowth of the optic canal. Timely surgical intervention is critical for preventing permanent vision loss in affected patients. This review summarizes the available literature on the surgical techniques of endoscopic endonasal optic nerve decompression (EEOND) and the transcaruncular approach in osteopetrosis patients. EEOND is a minimally invasive technique that offers excellent visualization, advanced instrumentation, and access to the optic nerve through the nasal corridor and requires mastering the anatomy of the sphenoid bone to achieve success. However, challenges arise from the dense and brittle nature of the bone in osteopetrosis, complicating the procedure. The transcaruncular approach provides a targeted route to the medial optic canal with minimal external scarring. However, its limited scope may not suffice for extensive decompression in severe cases. Early surgical intervention correlates with better visual outcomes, particularly in pediatric patients who are at higher risk for rapid progression of vision loss. Integrating advanced imaging and hybrid surgical techniques may enhance decompression efficacy. In conclusion, both EEOND and the transcaruncular approach are valuable for managing optic nerve compression in osteopetrosis, each with distinct advantages and limitations. Ongoing advancements in surgical techniques and a multidisciplinary approach are essential to optimize patient outcomes.

## Introduction

### Overview of Osteopetrosis

Osteopetrosis is a rare genetic disorder characterized by impaired osteoclast function, leading to defective bone resorption. In normal bone homeostasis, osteoblasts and osteoclasts work in opposition to maintain adequate bone remodeling. Osteoclasts balance bone deposition by osteoblasts through resorption. In osteopetrosis, mutations affecting proton pumps, carbonic anhydrase enzymes, or chloride channels reduce osteoclast numbers or render them ineffective. As a result, bones become abnormally dense, unorganized, and brittle. 1 Patients with osteopetrosis often present with characteristic radiographic findings such as osteosclerosis, increased cortical thickness, and decreased medullary canal diameter. 1 2

### Clinical Manifestations and Complications

The clinical manifestations of osteopetrosis vary depending on the pattern of inheritance. In autosomal recessive osteopetrosis (ARO), patients may present with hepatosplenomegaly, failure to thrive, and recurrent infections secondary to bone marrow failure. 2 3 In contrast, the autosomal dominant form (osteopetrosis tarda) is typically milder and often presents in childhood or early adulthood with symptoms such as fractures, hearing loss, and visual impairment. The failure to resorb bone leads to bony overgrowth, which can result in craniofacial abnormalities, such as synostosis and plagiocephaly, as well as compressive neuropathies, particularly affecting cranial nerves II and VIII. Optic nerve compression can result in progressive vision loss, and patients with ARO are particularly at risk for blindness due to compressive optic neuropathy. 1 2 4

### Compressive Neuropathy of the Optic Nerve

In osteopetrosis, vision loss is primarily due to compressive neuropathy, which occurs due to bony overgrowth around the optic canal, exerting pressure on the optic nerve. This pressure disrupts blood flow and axonal transport, leading to ischemia and progressive optic atrophy. 4 5 Additionally, the bony overgrowth can restrict cerebrospinal fluid (CSF) flow and cause further damage to the optic nerve. 2 6 The anatomy of the optic canal, particularly its narrow intracanalicular portion, predisposes the optic nerve to compression. The optic canal is bordered medially by the body of the sphenoid bone and sphenoid sinus and laterally by the lesser sphenoid wing. Its roof, which is only 1 to 3 mm thick, is particularly vulnerable to bony overgrowth. 2 6

### Optic Nerve Decompression

Optic nerve decompression is a critical intervention for patients with compressive optic neuropathy due to osteopetrosis. 6 The goal of the procedure is to relieve pressure on the optic nerve by removing the overgrown bone, thereby restoring blood flow and potentially reversing vision loss. 7 The degree of visual recovery depends heavily on the timing of the intervention as early decompression is associated with better outcomes. 6 However, there are no clear guidelines on the optimal timing of surgery, and research on nontraumatic optic nerve decompression, particularly in osteopetrosis, is limited. 2

## Objective

This review aims to summarize the available literature on the surgical techniques of endoscopic endonasal and transcaruncular optic nerve decompression in patients with osteopetrosis.

## Endoscopic Endonasal Approach: Surgical Techniques

### Surgical Methodology

The endoscopic endonasal optic nerve decompression (EEOND) is a minimally invasive approach that allows access to the optic nerve by navigating through natural anatomical corridors such as the sphenoid and ethmoid sinuses, bypassing the need for large external incisions and minimizing the risk of scarring. 7

First, a thorough preoperative imaging assessment using high-resolution CT or MRI scans to delineate the patient's anatomy is needed. 8 9 Once in the operating room, the procedure typically starts with a sphenoethmoidectomy. This involves removing portions of the ethmoid and sphenoid sinuses to expose the optic canal and surrounding structures, providing the surgeon with a clear line of sight to the optic nerve. If an Onodi cell is present—a posterior ethmoid air cell that often encroaches on the sphenoid sinus—it must be carefully opened to avoid damaging the optic nerve, which lies in close proximity. 10 11

After the sphenoid sinus has been opened, the optic nerve and the internal carotid artery are identified within the superolateral wall of the sphenoid sinus. Extreme care must be taken during this step due to the complex anatomy and the close relation of the optic nerve and the internal carotid artery. 5 The opticocarotid recess (OCR), bordered by the optic nerve superiorly and the carotid artery inferiorly, serves as an important anatomical landmark, helping to orient the surgeon to the optic nerve's location relative to the carotid artery. Identifying the OCR allows for a safe route to decompress the nerve. 7 10 11

Then, the mucosa overlying the optic canal is dissected, and the bone covering the canal is carefully drilled away using a diamond burr. Continuous water irrigation is necessary at this step to prevent drilling-induced thermal damage to the optic nerve. 7 The goal is to thin the bony covering of the optic nerve until only a thin layer of bone remains, which can then be gently fractured away, fully exposing the nerve. 7 10 11

Once the optic canal is sufficiently exposed, decompression of the nerve is performed. This may involve removing the annulus of Zinn—the fibrous ring surrounding the optic nerve at the apex of the orbit—or, in some cases, incising the dural sheath of the optic nerve to relieve pressure caused by CSF accumulation or constriction within the bony canal. There is an ongoing debate within the surgical community about the risks and benefits of dural incision; while it can relieve intracanal pressure, it also carries an increased risk of CSF leakage. 10 11

### Challenges in Osteopetrosis

Osteopetrosis presents specific challenges in optic nerve decompression due to the excessive bone density characteristic of the disease. 5 Unlike other pathologies that cause optic nerve compression, the bone in osteopetrosis is not only thick but also brittle and poses a challenge to remove, increasing the technical difficulty of the procedure. 7 The sclerotic nature of the bone requires the use of high-powered drills and specialized burrs, which increases the risk of thermal injury to the nerve or adjacent structures. Additionally, the distorted anatomy in osteopetrosis can obscure crucial landmarks, making it rigorous for surgeons to navigate and increasing the risk of inadvertent injury to the internal carotid artery or other critical structures. 8 9

Furthermore, in some cases, the optic nerve can be severely encased by the bony overgrowth, making its visualization difficult, even with modern and advanced endoscopic techniques. This can result in incomplete decompression, which may leave the nerve at risk for continued compression and ongoing visual loss. Additionally, the thickened, high-density bone of the optic canal in osteopetrosis patients can obscure fine anatomical details on CT scans, decreasing the yield of preoperative imaging for surgical planning. 7 9

The challenge is even more pronounced in pediatric patients, where access through surgical corridors is complicated by the smaller anatomy and incomplete development of the sinuses. In young children, the sinuses are not fully pneumatized, which means that surgeons must often drill through solid bone to reach the optic canal. 11 This increases the risk of damaging adjacent structures, such as the internal carotid artery or the dura mater, potentially resulting in an increased risk for significant complications.

Consequently, surgeons must be highly, trained, skilled, and experienced in advanced endoscopic skull base techniques to safely navigate the distorted anatomy and dense bone encountered in osteopetrosis. Intraoperative navigation systems and real-time imaging have become important tools in ensuring that the procedure is performed safely and effectively. These systems allow for precise mapping of the patient's unique anatomy, helping surgeons avoid critical structures and achieve a more complete decompression of the optic nerve. 5

## Transcaruncular Approach: Surgical Techniques

### Surgical Methodology

The transcaruncular approach offers an alternative route for optic nerve decompression, particularly in cases where the compression is more localized to the medial orbit. 12 This approach is less invasive in terms of external visibility, as the incision is made through the conjunctiva of the medial canthus, avoiding any external facial scarring. The transcaruncular approach provides direct access to the medial aspect of the optic canal through a relatively short surgical corridor, making it a useful option for patients with more localized optic nerve compression. 13 14

The procedure begins with a vertical incision in the conjunctiva, extending approximately 10 mm superiorly and inferiorly from the caruncle. The conjunctival tissue is dissected to expose the periosteum of the medial orbital wall. Care must be taken to avoid damaging the medial rectus muscle, which lies in close proximity to the surgical site. The periosteum is incised, and a subperiosteal dissection is performed to access the optic canal. 13 14

Once the medial orbital wall is exposed, the bone overlying the optic canal is carefully removed using a drill or a thin periosteal elevator.

At the end of the procedure, the conjunctiva and caruncle are closed with fine sutures to ensure proper healing and minimize the risk of postoperative complications such as conjunctival scarring or infection. Postoperatively, patients are monitored for signs of infection, bleeding, or diplopia, which can result from damage to the medial rectus muscle during surgery. 13 14

### Application in Osteopetrosis

The transcaruncular approach is particularly well-suited for cases of optic nerve compression that are more localized to the medial aspect of the optic canal, such as those seen in osteopetrosis. 15 However, the dense, sclerotic bone characteristic of osteopetrosis makes the procedure more technically demanding. The bone overlying the optic canal may need to be thinned before it can be safely removed, and the risk of fracturing the surrounding bone is increased due to the brittle nature of the bone in osteopetrosis patients. Additionally, the proximity of the medial rectus muscle to the surgical site increases the risk of postoperative complications such as diplopia or strabismus.

Despite these challenges, the transcaruncular approach offers a minimally invasive option for optic nerve decompression, particularly in patients who are not candidates for more extensive surgical interventions. However, due to the tight surgical access provided by the transcaruncular approach, the decompression is typically limited to the medial and superior aspects of the optic canal. 7

## Discussion

### Comparison of Approaches

Both EEOND and the transcaruncular approach offer distinct advantages and face particular challenges in managing optic nerve compression in osteopetrosis. EEOND is a minimally invasive technique that provides excellent visualization of the optic nerve and surrounding structures, particularly in the central skull base. The use of endoscopic tools allows for precision and a wide field of view without the need for external incisions, reducing the risk of visible scarring and promoting faster recovery times. This makes EEOND particularly suitable for cases where access to the central skull base and sphenoid sinus is crucial, and where the optic nerve is severely compressed. 16

However, the increased bone density in osteopetrosis often limits the extent of bone removal achievable during EEOND, particularly in cases where the bone is abnormally thick or sclerotic. This can reduce the effectiveness of decompression, especially if the optic canal remains partially occluded. Furthermore, the dense bone complicates the drilling process, increasing the likelihood of intraoperative complications such as thermal injury to the optic nerve, inadvertent damage to adjacent critical structures like the internal carotid artery, or CSF leaks. In pediatric patients, where the skull base is still developing and the sinuses are incompletely pneumatized, these challenges are further amplified, necessitating the use of smaller instruments and more cautious surgical planning. 1 7 11 For instance, in the case described by Yang et al, intraoperative neuronavigation was critical for navigating the undeveloped anatomy and ensuring safe and effective decompression. 15

As shown in the case reported by Yang et al, a 22-month-old female with ARO and severe congenital optic canal stenosis underwent bilateral EEOND. Despite the absence of sphenoid sinus pneumatization, the procedure allowed for effective decompression of the optic canals, resulting in significant improvements in visual evoked potential amplitudes and behavioral responses postoperatively. 15 Similarly, Berhouma et al reported on 11 patients with various conditions, including osteopetrosis, who underwent EEOND for optic canal decompression. Over half of these patients experienced improved visual acuity within 6 months, highlighting the procedure's ability to restore vision in complex anatomical scenarios. 8 These cases demonstrate EEOND's utility in addressing severe and bilateral optic nerve compression, even under challenging anatomical conditions.

The transcaruncular approach, by contrast, provides a more targeted pathway to the medial orbital wall and optic canal through a conjunctival incision. This technique avoids the potential for extensive bone drilling and CSF leaks associated with EEOND, and it offers a minimally invasive route with less visible scarring. As demonstrated in the case reported by Medsinge et al, a 6-month-old infant with ARO presented with progressive vision loss due to narrowing of the optic canals. The patient underwent bilateral optic canal decompression via the transcaruncular approach, which involved careful medial orbital wall decompression through a conjunctival incision to access and decompress the optic canals. Postoperatively, the patient exhibited improvement in visual acuity, reduced nystagmus, and maintained open optic canals on follow-up imaging. This case underscores the suitability of the transcaruncular approach for localized medial optic canal compression, particularly in pediatric patients where minimally invasive techniques are preferred. 17 However, the transcaruncular approach is more limited in terms of the extent of decompression achievable, as it is primarily used to access the medial optic canal. Additionally, because the incision is made in the conjunctiva, there is an increased risk of injury to the medial rectus muscle, which can result in postoperative diplopia, gaze palsy, or restricted eye movement. In osteopetrosis, where bone overgrowth can be extensive and unpredictable, this approach may be insufficient to achieve full decompression, particularly in cases of severe optic nerve compression. 17

### Timing and Outcomes of Surgery

One of the key factors influencing the success of optic nerve decompression is the timing of the intervention. Early surgical intervention is associated with superior visual outcomes, as it prevents further ischemic damage to the optic nerve caused by prolonged compression. Several case reports and retrospective studies have demonstrated that early decompression can halt the progression of visual loss and, in some cases, even reverse some degree of optic nerve damage. For example, in nontraumatic cases of optic neuropathy, such as those caused by osteopetrosis, patients who undergo surgery within the first few weeks of symptom onset are more likely to experience improvements in visual acuity compared to those who delay treatment. However, the exact window of optimal timing remains unclear, as there are no standardized guidelines specific to osteopetrosis. 16

In pediatric patients, the timing of surgery can be even more critical. Children with osteopetrosis are at higher risk of early-onset blindness due to the rapid progression of optic nerve compression. Thus, timely intervention is crucial to preserve vision in these patients. However, the risks associated with early surgery, such as anesthesia complications and the technical challenges of operating on a developing skull base, must be carefully weighed against the potential benefits. Long-term studies following pediatric patients who have undergone optic nerve decompression are limited, and further research is needed to better understand the ideal timing for surgical intervention in this population. 7

### Role of Hybrid Techniques

Given the limitations of both EEOND and the transcaruncular approach, there is growing interest in the development of hybrid techniques that combine the strengths of both approaches to optimize decompression. For example, a hybrid technique might involve using the transcaruncular approach to access the medial orbit and perform decompression in that area, followed by EEOND to access the central skull base and sphenoid sinus. This would allow for more comprehensive decompression of the optic nerve while minimizing the risks associated with each individual approach. While hybrid techniques have not yet been widely adopted in clinical practice, preliminary case reports suggest that they may offer a promising solution for patients with complex anatomical challenges, such as those seen in osteopetrosis. 18

### Future Directions in Research

Advancements in endoscopic technology, such as the development of high-definition cameras, robotic-assisted surgery, and enhanced surgical instruments, hold great promise for improving the outcomes of optic nerve decompression in osteopetrosis. Additionally, the integration of intraoperative neuro-navigation and real-time imaging could provide surgeons with a more accurate understanding of the anatomy they are working with, allowing for safer and more effective decompression.

Another important area of future research is the long-term follow-up of patients who undergo optic nerve decompression for osteopetrosis. While short-term outcomes are generally favorable, particularly in terms of visual acuity improvement, there is limited data on the long-term stability of these results. As mentioned earlier, regrowth of dysplastic bone is a common issue in osteopetrosis, and repeat surgeries may be necessary to maintain the decompression achieved by the initial procedure. Long-term studies could help to identify predictors of success and recurrence, as well as inform the development of preventive strategies to reduce the need for reoperation.

In addition, future studies should focus on understanding the optimal timing of surgery, especially in pediatric patients, and determining how to balance the risks and benefits of early intervention. Multicenter trials with larger sample sizes are needed to establish evidence-based guidelines for the timing of optic nerve decompression in osteopetrosis.

### Role of Multidisciplinary Teams

Given the complexity of osteopetrosis and its associated complications, a multidisciplinary approach is often necessary to optimize patient outcomes. Neurosurgeons, otolaryngologists, ophthalmologists, and anesthesiologists must work together to carefully plan and execute these intricate procedures. Preoperative planning is critical, and collaboration between these specialties can help anticipate and address potential challenges during surgery. The role of postoperative rehabilitation, including ophthalmologic and neuro-ophthalmologic follow-up, is equally important to monitor for complications, assess visual recovery, and ensure that any recurrence of bony overgrowth is promptly addressed. 13 15

## Conclusion

Optic nerve decompression is a challenging yet essential intervention in the management of osteopetrosis-related compressive optic neuropathy. Both endoscopic endonasal and transcaruncular approaches offer valuable techniques, each with its advantages and limitations. EEOND allows for comprehensive visualization and decompression of the optic nerve, particularly in the central skull base, but is limited by the dense, sclerotic bone of osteopetrosis. The transcaruncular approach provides a minimally invasive option for targeted decompression of the medial orbit, but its scope is more limited, particularly in severe cases of optic nerve compression.

As surgical techniques and technologies continue to advance, there is great potential for improving outcomes in patients with osteopetrosis. Hybrid approaches, robotic-assisted surgery, and enhanced imaging modalities may help overcome the current limitations of these techniques and offer better long-term results. Future research should focus on the development of evidence-based guidelines for the timing of surgery, long-term follow-up of patients, and the integration of multidisciplinary care to ensure the best possible outcomes for these complex cases.
